# Geohistorical dataset of ten plant species introduced into Occitania (France)

**DOI:** 10.3897/BDJ.10.e76283

**Published:** 2022-03-28

**Authors:** Morgane Claudel, Emilie Lerigoleur, Cécile Brun, Sylvie Guillerme

**Affiliations:** 1 LTSER Zone Atelier Pyrénées-Garonne, CNRS, Université de Toulouse, Castanet Tolosan, France LTSER Zone Atelier Pyrénées-Garonne, CNRS, Université de Toulouse Castanet Tolosan France; 2 UMR 5602 GEODE CNRS-UT2J, Toulouse, France UMR 5602 GEODE CNRS-UT2J Toulouse France; 3 Université de Nantes, Nantes, France Université de Nantes Nantes France

## Abstract

**Background:**

The original dataset presented here is the result of the first near-exhaustive analysis performed on historical data concerning ten plant species introduced in and around Occitania (south-western France) since 1651. Research was carried out on the following species: *Alnusincana*, *Buddlejadavidii*, *Castaneasativa*, *Helianthustuberosus*, *Impatiensglandulifera*, *Prunuscerasifera*, *Prunuslaurocerasus*, *Reynoutriajaponica*, *Robiniapseudoacacia* and *Spiraeajaponica*.

The data file contains 199 occurrence data exclusively based on historical observations and records made between 1651 and 2004 that were retrieved from 111 of the 640 literary sources consulted. All the records are associated with a year and 61% of them have associated spatial coordinates. Initially, the EI2P-VALEEBEE research project focused on the introduction of these species into Occitania (95 occurrences, 47.7%), but mentions found of introductions beyond this territory - mainly in metropolitan France - are also reported.

The creation of this dataset involved five stages: (1) selection of species, (2) consultation of historical sources, (3) recording of occurrences in the dataset, (4) dataset standardisation/enrichment and Darwin core mapping and (5) data publication. Quality controls were conducted at each step.

The dataset is available on the platform of the Global Biodiversity Information Facility (GBIF) at https://doi.org/10.15468/3kvaeh. It respects the internationally recognised FAIR Data Principles (Findable, Accessible, Interoperable and Reusable).

**New information:**

The dataset will be progressively enriched by new data during the EI2P-VALEEBEE research project and future projects on invasive plant species conducted by the team.

## Introduction

The introduction of alien species into a given region may be intentional, for ornamental, horticultural or agricultural purposes, but more often, it is involuntary (Pyšek et al. 2009; Laporte-Cru and Aniotsbéhère 2010). Whatever the case, invasive alien species have multiple consequences on biodiversity worldwide, as do the destruction of natural habitats, pollution, overuse of resources and climate change (Pimentel 2011; Bellard et al. 2012; Verma et al. 2020). Introduced species, if and when they become invasive, induce multiple consequences, direct and/or indirect, affecting the native species, the functioning of natural habitats and the services provided by ecosystems, as well as economic activities and human health (IUCN French Committee 2018). For some authors, the invasive character of an alien plant is linked to both the environment and the species (Rejmanek et al. 2005; Richardson and Pysek 2006). Therefore, it is impossible for an observation made in a given place to be generalised to all environments. The history of such introductions is also a criterion that it is important to consider when attempting to understand the invasion dynamics. Introduction patterns and historical factors resulting in the presence of alien species in a given region could provide key information for risk management and the prevention of potentially harmful introductions (Dudeque Zenni 2014).

The data presented here are derived from the EI2P-VALEEBEE project, co-funded by the Région Occitanie and the Maison des Sciences de l'Homme de Toulouse (cf. glossary of acronyms in Suppl. material 1). They combine two issues of major ecological, socio-cultural and economic concern: biological invasions and the decline of pollinator populations. The main objective of this project is to better identify the links between plant invasions and changes in ecosystem services (Vaz et al. 2017a; Pisani et al. 2021) so as to better understand the potential or constraints of invasive alien plants in connection with pollinators and try to apprehend invasive processes through the most systemic approach, by taking several dimensions into account (human, spatial, historical, ecological and ethological) and by carefully considering the practices, perceptions and representations that the various stakeholders in the territory have manifested over time (Vaz et al. 2017b). This systemic approach is also found in the project's method itself, since it brings together several disciplines and tools and also has a diachronic dimension over several time-scales. In carrying out the project, it was decided to focus on the Occitania Region in the south-western part of France and, more particularly, on two territories: the Pique Valley and the Oussouet Valley (Guillerme et al. 2020), both of which have not only a high level of plant diversity, but also a high rate of invasion.

The issue of alien plant invasions is complex and multifactorial. It includes an important geographical dimension, since the distribution of invasive alien plants is conditioned by the variations of an environment (Souty-Grosset et al. 2015) and it also includes a huge temporal dimension, since it is a process that takes place in the long term. In this perspective and as suggested by Renault et al. (2015), we intend to study the phenomenon in the most transversal and objective way possible to better understand and adapt our actions towards these species. On this basis, the EI2P-VALEEBEE project has chosen to combine the study of plant invasions with the process of decline of pollinators, which is also a multifactorial phenomenon with major ecological and socio-economic importance, since 70% of the plants used in the world for our food depend on pollination by insects (Klein et al. 2007). This decline is partly due to the fall in the quality and quantity of available melliferous resources (Vanbergen et al. 2017). However, some exotic species, considered as alien plants in Occitania, seem to have strong potential in terms of nectar production and melliferous resources.

The EI2P-VALEEBEE project aims to deepen our knowledge of the links between alien plant invasions and changes in ecosystem services, which are still poorly understood today (Drenovsky et al. 2012) and which could potentially change the way we look at alien species and our management strategies towards them. As Simberloff et al. (2012) point out in general, or Renault et al. (2021) more specifically for France, there is a need for general information on these species. Several databases documenting invasive alien species distributions currently exist (CABI: Invasive Species Compendium 2021, Global Invasive Species Database 2020, Global Register of Introduced and Invasive Species 2020, CABI: Invasive Species Compendium 2021). The history of the introduction of a non-native species into a region is linked to the first observations described and establishing it often requires cross-checking of all the information collected. It is a question of tracing the evolution and progression of these alien species from the date and place of their introduction and of noting their behaviour in our ecosystems according to the first observations that were recorded (Laporte-Cru and Aniotsbéhère 2010).

At the scale of Occitania and, more broadly, at the French national scale, our dataset provides novelty in the consideration of the temporal and geohistorical dimension of the invasion phenomenon as it records both observations and historical and literary mentions of the species studied.

## General description

### Purpose

The data file is the result of a geo-historical study conducted on the introduction and distribution of invasive plant species. Ten plant species were selected that can all be observed in the Pique and Oussouet Valleys. Some of them are considered as invasive, alien species. The study includes research on the introduction dates of the species studied, the locations of their introduction, their interest and past uses, the different human perceptions of them over time, activities that have impacted their local distribution, comments from authors and observers on their abundance and elements of the historical context of their introduction. Historical sources were consulted during 2020 in order to find the oldest elements concerning the ten species.

### Additional information

**Interest and use of the dataset**

Without a historical analysis, it is difficult to understand the current local distribution dynamics of invasive plant species, especially since some of them were introduced into Metropolitan French territory several centuries ago (Souty-Grosset et al. 2015). A major interest of this dataset is to provide historical depth and chronological elements for the understanding of the current distribution of these ten species at local scale. In this perspective, the dataset is relevant to:

- identify the different periods of introduction of the ten species studied;
- provide accurate data on the main introduction channels, such as the place where species were introduced (mainly ornamental gardens, thermal parks and private gardens, but, for some of them, also much wilder and more natural areas, such as road borders and forests);
- identify pathways of colonisation to understand the current localisation of each species;
- enable better understanding of why they were introduced, particularly thanks to information on their uses;
- provide elements of analysis to understand the temporal phases of species distributions.

The interest of the dataset is directly in line with the values of the EI2P-VALEEBEE project itself, the objective of which is to contribute to a better understanding of plant invasion processes in a transversal way (Renault et al. 2015). In this perspective, it can be useful for all local, national and international organisations involved in this issue with current ecological, economic and social implications.

## Project description

### Title

Geohistorical dataset of ten plant species introduced in Occitania (France)

### Personnel

Conceptualisation, M.C. and E.L.; methodology, M.C., E.L., C.B. and S.G.; investigation, M.C.; data validation, M.C. and E.L.; writing, review and editing, M.C., E.L., C.B. and S.G.; supervision, C.B. and S.G.; project administration, S.G.; funding acquisition, S.G.

### Study area description

The Oussouet Valley (Pyrenean foothills, Hautes-Pyrénées) and the Pique Valley (Haute-Garonne) in Occitania (South of France).

### Funding

- Région Occitanie - Appel à projets Recherche et Société(s) 2019
- Maison des Sciences de l'Homme et de la Société de Toulouse (MSH-T) APEX 2020

## Sampling methods

### Sampling description

The creation of this dataset involved a number of different stages: (1) selection of species, (2) consultation of historical sources, (3) recording of occurrences in the dataset, (4) dataset standardization/enrichment and Darwin core mapping and (5) data publication.

Step 1: Species selection.

Current field observations were made particularly in the two valleys selected in the south-west of France: the Pique Valley and the Oussouet Valley. These two territories present a high rate of plant invasions. Four exotic plant species were initially observed to provide some spatial coverage in these valleys: *Buddlejadavidii* Franch., 1887; *Impatiensglandulifera* Royle, 1833; *Reynoutriajaponica* Houtt., 1777 and *Spiraeajaponica* L.f., 1782. In accordance with the scientific needs of the EI2P-VALEEBEE project, six species (*Alnusincana* (L.) Moench, 1794; *Castaneasativa* Mill., 1798; *Helianthustuberosus* L., 1753; *Prunuscerasifera* Ehrh., 1784; *Prunuslaurocerasus* L., 1753 and *Robiniapseudoacacia* L., 1753) were added to the selection on the basis of the main relevant criteria (see glossary for acronyms in Suppl. material 1):

- a lack of reference data in the existing literature at the national and/or local level (CBNPMP, GBIF, IUCN, Catalogue of Life, INPN, Tela Botanica BDTFX, Baseflor DB- Ph. Julve, Invasive Species Compendium CABI ISC, Delivering alien invasive species in Europe, DAISIE);
- the distribution of the species in Occitania (Human observations, GBIF);
- their current status (Muller 2004, CBNPMP, GBIF, IUCN);
- the most frequently occurring species in the southwest of France (Planty-Tabacchi 1997);
- the current knowledge on their honey and nectar potentials (FranceAgriMer, ITSAP, Ministry of Agriculture and Food, SNHF, Astredhor and the VAL'HOR inter-professional association, in partnership with INRA, CNPAIM, GNIS and SBF).

Step 2: Consultation of historical sources.

For the consultation of historical sources, a funnel method was applied:

1. To begin, the existing sources of naturalist data were inventoried at the national level, such as flora (e.g. Flore de France, Coste 1906); seed catalogues (e.g. Andrieux and Vilmorin 1783); horticultural, botanical and beekeeping newspapers and magazines (e.g. Bulletins de la Société Centrale d'Apiculture 1856-1946, Bulletins de la Société Nationale de Protection de la Nature 1882-1888). This first national-scale inventory gave an idea of the presence of the ten species in France during the different periods of history. It also allowed the written sources containing botanical elements on a smaller scale to be identified.
2. The second stage was the analysis of literary sources at the regional level (Occitania). Local herbaria (e.g. Bordere 1871-1872), regional flora (e.g. Philippe 1859), botanical magazines and newspapers of the region (e.g. Société d'Horticulture 1854-1921) were inventoried and analysed. The seed merchants and horticulturists located in the region were also inventoried to explore their seed catalogues (e.g. Catalogue des plantes vivaces et d'extérieur, Bonamy Frères 1874) and potentially find elements on the colonisation routes of the species. In addition, efforts were made to be as exhaustive as possible by consulting archival institutions at the departmental level (Departmental Archives of Hautes-Pyrénées, Departmental Archives of Haute-Garonne). The archived documents consulted were of all kinds: some known botanists' collections (e.g. Bouget 1901-1948), invoices related to seed orders, documents on the construction of natural parks (Natural Park of the Pyrenees collection, Aymonin 1975) and thermal gardens (“Projet d’aménagement du Parc des Quinconces”, Chevalier 1902), together with herbaria, letters and notes. Examining these references for specific information on introduced exotic species and their spatial location was innovative.
3. Once enough regional historical sources had been analysed, it was possible to focus on the scale of our study sites. The main idea was to study historical sources on the scale of the municipalities located in the Pique and the Oussouet Valleys. For this purpose, municipal archives (e.g. Municipal archives of Bagnères-de-Luchon, Municipal archives of Tarbes) were also consulted, taking care to identify the potential pathways of introduction on a very local scale in advance, to facilitate the search in archived articles. As local data sources, archival records enabled consultation of community monographs that presented a chapter on the local flora (e.g. Barèges monograph, Rondou 1907). Finally, newspapers associated with the municipalities (e.g. Revue de Comminges, Société des Études du Comminges 1885-2004) and local herbaria (e.g. Ramond de Carbonnières 1755-1825), offered information on the flora that was much more local and precise.

Throughout the research process, key informants, having good knowledge of the study areas and their backgrounds, contributed information and advice on valuable literary sources, allowing the research to best fit the study areas and to be as complete as possible. It should also be noted that, in order to consult sources of all kinds, as soon as we felt that a literary source could potentially contribute elements on one of the ten species, we consulted it, even if, at first sight, it had no connection with botany (e.g. the recipe book of La Varenne 1651).

As a result, 640 literary sources were consulted during this step. Amongst these, 111 (17.3%) provided information on the introduction and colonisation of the ten species over time (Suppl. material 3). It must be understood that consulting historical sources was one of the most important steps, not only in the creation of this dataset, but also for its future updates, because this work made it easier to identify potential sources of information and distinguish them from blind alleys.

Step 3: Occurrences recorded in the data file.

Each time a species was mentioned in the historical literature consulted, it was recorded in an occurrence data file created in LibreOffice Calc (spreadsheet programme). The file format is OpenDocument Spreadsheet (.ods). When recording an occurrence, attention was paid to the vernacular and scientific synonyms used in the historical literature. We recorded the occurrences for which the historical name is currently identified as a synonym in the Catalogue of Life, INPN and ISSG, but also according to the number of elements in the bibliographic source that allowed the taxon to be identified as such: photographic representation, image or plate of the species, precise description, mention of other known scientific and vernacular names. In the data file, a maximum of elements mentioned by the author were recorded: the synonym cited; the reference code from the French taxonomic referential TAXREF (https://inpn.mnhn.fr/programme/referentiel-taxonomique-taxref?lg=en); the bibliographic reference in which the mention of the species was found; the date of observation of the species or, failing that, the date of its mention; the names of the observers and authors; the type of source; the description of the location as soon as it was mentioned; the species' spatial coverage and abundance; its minimum and maximum altitudes; any comments by the author about the species, the location of observation or mention of the species; the nearest town/village; and the latitude and longitude coordinates. In addition, each element concerning the literature source was recorded in the same software (LibreOffice Calc): bibliographic reference number (identifier), name(s) of the author(s), title, year of publication, collection and publisher if they were mentioned, as well as the city of publication, the name of the journal (if the source was an article), the URL if accessible or the reference of the archive document with the location of the archive institution, the call number and the series.

Step 4: Dataset standardisation/enrichment and Darwin Core mapping.

The Darwin Core Standard (Wieczorek et al. 2012) "offers a stable, straightforward and flexible framework for compiling biodiversity data from varied and variable sources. (...) This standardization not only simplifies the process of publishing biodiversity datasets, it also makes it easy for users to discover, search, evaluate and compare datasets as they seek answers to today’s data-intensive research and policy questions." (Source: https://www.gbif.org/darwin-core).

Each column header of the occurrence spreadsheet was searched for an equivalent term in the Darwin Core quick reference guide (https://dwc.tdwg.org/terms/). We also chose the Identification History extension (https://tools.gbif.org/dwca-validator/extension.do?id=dwc:Identification) to manage synonyms of taxon names as they were cited in the literature consulted.

The geographic data (longitude, latitude, WGS84 datum) were structured in two ways: 1) geographic coordinates with an accuracy of 500 metres for occurrences whose locality was precisely identified in the literary source and 2) geographic coordinates with an accuracy of 10,000 metres for occurrences whose literary source mentioned only the name of the municipality (https://www.geonames.org/). For each occurrence, the Darwin core terms “country”, “province”, “county”, “municipality” and “locality” were assigned as far as possible from the elements of the literary sources.

Step 5: Data publication.

For the publication on the GBIF platform, the Integrated Publishing Toolkit (IPT (gbif.org)) of the GBIF was used to fill out the metadata and to generate the Darwin Core Archive. The dataset is available on the GBIF platform at https://doi.org/10.15468/3kvaeh (Claudel et al. 2021). The dataset now respects the FAIR Data Principles (Findable, Accessible, Interoperable and Reusable) defined by Wilkinson et al. (Wilkinson 2016). Table 1 summarises the FAIRness assessment criteria used to make the dataset FAIR.

### Quality control

Several quality controls were implemented. First, data cleaning and corrections were performed with proofreading by a third party and the use of pivot tables to check data integrity. Harmonisation and standardisation of content were also necessary to allow and facilitate the mapping with Darwin Core terms. The latter made it possible to identify new additional information such as nomenclatural code, coordinate uncertainty, geodetic datum, georeference source, licence etc. As many standards as possible were chosen to describe country codes (ISO 3166-1-alpha-2), municipality names and their geographic coordinates (geonames.org), taxon scientific names (TAXREF v.13.0 - 2019-12-06) and taxon ID from several sources (GBIF, IUCN, Catalogue of Life, IPNI, INPN, Tela Botanica BDTFX). Finally, we used a GIS tool (QGIS 3.10 LTR) to check the geographic coordinates of occurrences.

## Geographic coverage

### Description

**Figure 1.**
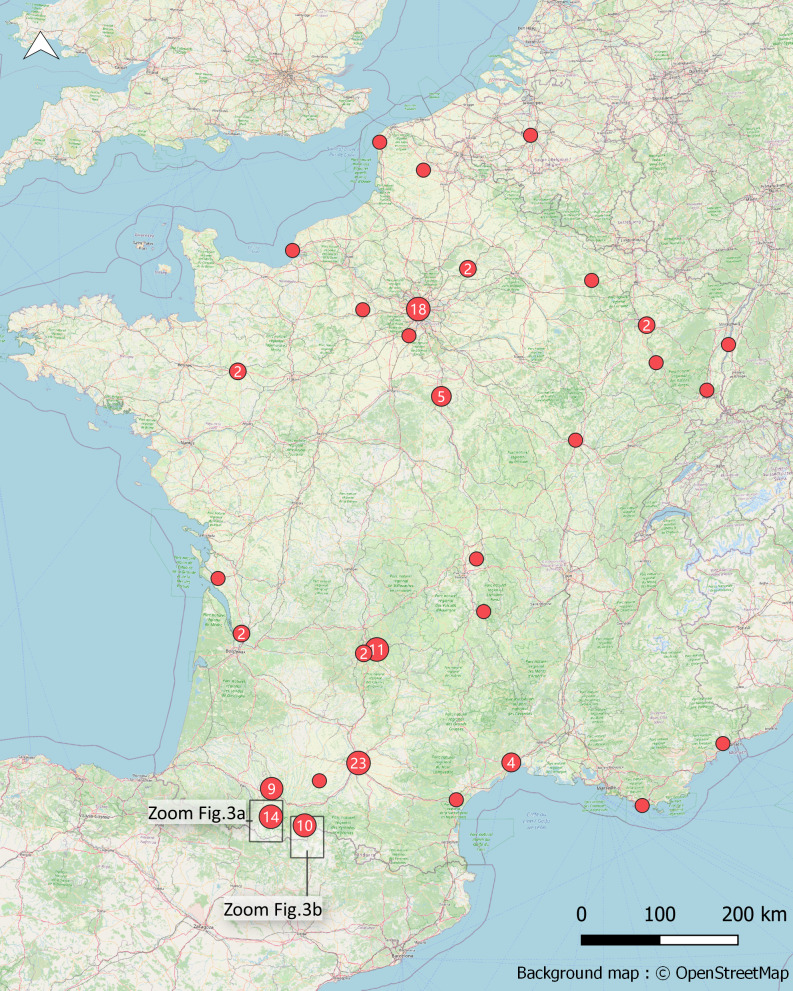
Spatial location of the 122 occurrences having data associated with coordinates.

**Figure 2.**
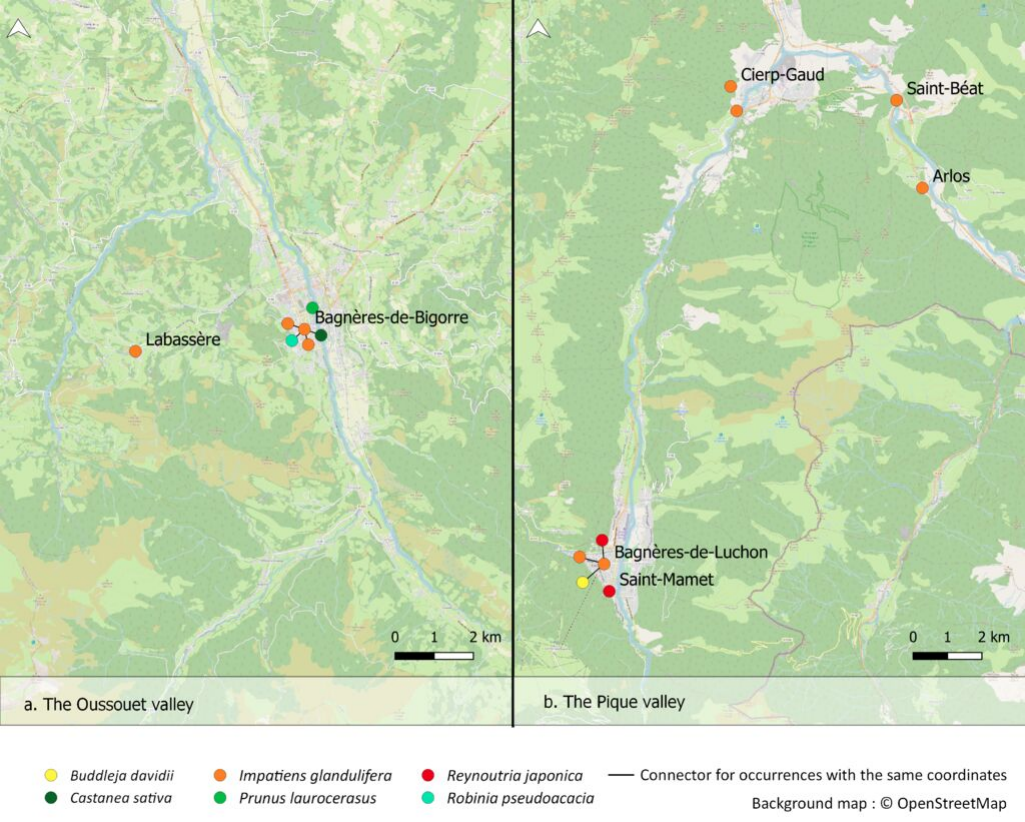
Focus on (**a**) the Oussouet Valley and (**b**) the Pique Valley.

Within the framework of this geohistorical study, we consulted the information on the introductions and distributions of target species existing in archival sources regarding the Oussouet Valley (Pyrenean foothills, Hautes-Pyrénées) and the Pique Valley (Haute-Garonne), in Occitania (95 occurrences, 47.7%). When information concerning other French or European territories: other parts of France (50.8%), Belgium (1%) or the UK (0.5%), was found in the documents consulted, it was also recorded. This explains the European geographical coverage. Of the 199 occurrences, only 122 (61%) occurrences are precisely geolocated (municipality or 500 metre buffer). Fig. 1 shows the geolocation of these 122 occurrences and Fig. 2a and Fig. 2b focus on the occurrences found in the Oussouet Valley and the Pique Valley.

### Coordinates

 and 42°5'52.8''N and 59°15'57.6''N Latitude Latitude; 8°36'46.8''W Longitude and 8°26'16.8''E Longitude Longitude.

## Taxonomic coverage

### Description

Ten plant species were studied: *Alnusincana* (L.) Moench, 1794; *Buddlejadavidii* Franch., 1887; *Castaneasativa* Mill., 1798; *Helianthustuberosus* L., 1753; *Impatiensglandulifera* Royle, 1833; *Prunuscerasifera* Ehrh., 1784; *Prunuslaurocerasus* L., 1753; *Reynoutriajaponica* Houtt., 1777; *Robiniapseudoacacia* L., 1753 and *Spiraeajaponica* L.f., 1782. The scientific names of the ten species comply with the national taxonomic and nomenclatural reference system for fauna, flora and fungi in metropolitan France and overseas: TAXREF v.13 (Gargominy et al. 2019). The database contains 199 records of these ten plant species. They are all angiosperms and belong to seven orders, eight families and nine genera (Table 2), classified according to APG IV (2016).

For the purposes of the historical study, all the Latin synonyms, identified and validated by the national taxonomic and nomenclatural reference frame TAXREF v.13 related to the ten taxa studied, were considered. During the analysis of historical documents, we also collected all vernacular synonyms as soon as they were associated with a validated Latin synonym (Suppl. material 2); they will help to enrich the current vocabulary designating these species.

## Temporal coverage

**Data range:** 1651-1-01 – 2004-1-01.

### Notes

The geohistorical database includes observation and record data for the ten species from 1651 until 2004, collected in 2020. The objective was to cover the different periods of introduction of these ten species in Occitania. We consulted literature dating from the 17th century, a period during which some of the species studied seem to have been introduced in France (*Helianthustuberosus*, *Prunuscerasifera*, *Prunuslaurocerasus* and *Robiniapseudoacacia*).

**Figure 3.**
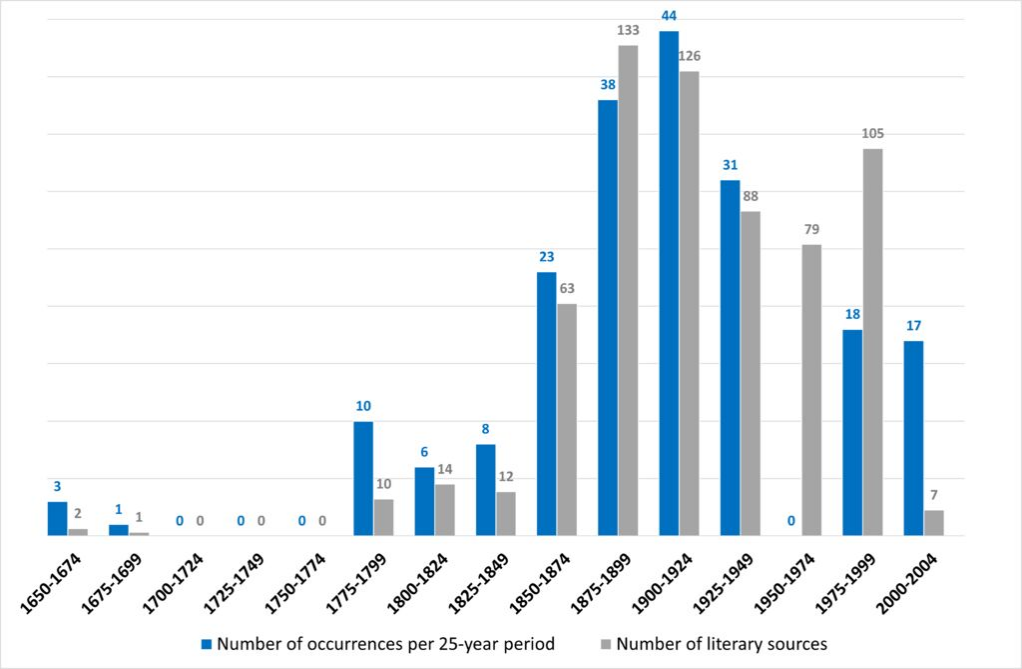
Number of occurrences found and number of literary sources consulted per 25-year period.

The number of collected occurrences increased from around 1800 until 1950, which can be explained by the introduction of four of the exotic species into Metropolitan France in the 19th century: *Buddlejadavidii*, *Impatiensglandulifera*, *Reynoutriajaponica and Spiraeajaponica* and also by an increase in the number of historical sources relating to botany and horticulture, which facilitated the identification of mentions or observations of the species studied. Therefore, 75% of the historical collected data dates from about 1800 to 1950 (Fig. 3). The lack of data for the 1950-1974 period could reflect the significant decrease in the number of naturalists’ records during this post-war period.

## Usage licence

### Usage licence

Other

### IP rights notes

Creative Commons Attribution (CC-BY) 4.0 License

## Data resources

### Data package title

dwca-ei2p_exotic_plants_geohistorical_occurrences_database-v1.4.zip

### Resource link

https://doi.org/10.15468/3kvaeh

### Number of data sets

2

### Data set 1.

#### Data set name

Darwin Core Archive Geohistorical plants occurrences database (occurrence.txt)

#### Data format

Darwin Core Archive format

#### Number of columns

50

#### Character set

UTF-8

#### Download URL

http://ipt.gbif.fr/archive.do?r=ei2p_exotic_plants_geohistorical_occurrences_database&v=1.4

#### Data format version

1.4

#### Description

The Darwin Core Standard (DwC) was used to offer a "stable, straightforward and flexible framework for compiling biodiversity data from varied and variable sources" (https://www.gbif.org/en/darwin-core). All column labels and descriptions are from https://dwc.tdwg.org/terms/.

**Data set 1.** 

Column label	Column description
id	Same as occurrenceID: An identifier for the Occurrence (as opposed to a particular digital record of the occurrence). In the absence of a persistent global unique identifier, construct one from a combination of identifiers in the record that will most closely make the occurrenceID globally unique. This is the primary key of this table.
type	The nature or genre of the resource.
modified	The most recent date-time on which the resource was changed.
language	A language of the resource.
licence	A legal document giving official permission to do something with the resource.
rightsHolder	A person or organisation owning or managing rights over the resource.
institutionID	An identifier for the institution having custody of the object(s) or information referred to in the record.
institutionCode	The name (or acronym) in use by the institution having custody of the object(s) or information referred to in the record.
datasetName	The name identifying the dataset from which the record was derived.
ownerInstitutionCode	The name (or acronym) in use by the institution having ownership of the object(s) or information referred to in the record.
basisOfRecord	The specific nature of the data record. Recommended best practice is to use the standard label of one of the Darwin Core classes.
occurrenceID	An identifier for the Occurrence (as opposed to a particular digital record of the occurrence). In the absence of a persistent global unique identifier, construct one from a combination of identifiers in the record that will most closely make the occurrenceID globally unique.
occurrenceRemarks	Comments or notes about the Occurrence.
recordedBy	A list (concatenated and separated) of names of people, groups or organisations responsible for recording the original Occurrence. The primary collector or observer, especially one who applies a personal identifier (recordNumber), should be listed first.
occurrenceStatus	A statement about the presence or absence of a Taxon at a Location. Recommended best practice is to use this controlled vocabulary: http://rs.gbif.org/vocabulary/gbif/occurrence_status.xml
associatedReferences	A list (concatenated and separated) of identifiers (publication, bibliographic reference, global unique identifier, URI) of literature associated with the Occurrence.
eventDate	The date-time or interval when the event was recorded. Not suitable for a time in a geological context. Recommended best practice is to use an encoding scheme, such as ISO 8601:2004(E).
year	The four-digit year in which the Event occurred, according to the Common Era Calendar.
eventRemarks	Comments or notes about the Event.
locationID	An identifier for the set of location information (data associated with dcterms:Location). May be a global unique identifier or an identifier specific to the dataset.
continent	The name of the continent in which the Location occurs. Recommended best practice is to use a controlled vocabulary such as the Getty Thesaurus of Geographic Names.
countryCode	A unique (preferably globally-unique) identifier for the taxon represented in the row. Recommended best practice is to use ISO 3166-1-alpha-2 country codes: http://rs.gbif.org/vocabulary/iso/3166-1_alpha2.xml
stateProvince	The name of the next smaller administrative region than country (state, province, canton, department, region etc.) in which the Location occurs. Recommended best practice is to use a controlled vocabulary such as the Getty Thesaurus of Geographic Names.
county	The full, unabbreviated name of the next smaller administrative region than stateProvince (county, shire, department etc.) in which the Location occurs. Recommended best practice is to use a controlled vocabulary such as the Getty Thesaurus of Geographic Names.
municipality	The full, unabbreviated name of the next smaller administrative region than county (city, municipality etc.) in which the Location occurs. Do not use this term for a nearby named place that does not contain the actual location. Recommended best practice is to use a controlled vocabulary such as the Getty Thesaurus of Geographic Names.
locality	The specific description of the place. Less specific geographic information can be provided in other geographic terms (higherGeography, continent, country, stateProvince, county, municipality, waterBody, island, islandGroup). This term may contain information modified from the original to correct perceived errors or to standardise the description.
verbatimLocality	The original textual description of the place.
minimumElevationInMetres	The lower limit of the range of elevation (altitude, usually above sea level), in metres.
maximumElevationInMetres	The upper limit of the range of elevation (altitude, usually above sea level), in metres.
locationAccordingTo	Information about the source of this Location information. Could be a publication (gazetteer), institution or team of individuals.
decimalLatitude	The geographic latitude (in decimal degrees, using the spatial reference system given in geodeticDatum) of the geographic centre of a Location. Positive values are north of the Equator, negative values are south of it. Legal values lie between -90 and 90, inclusive.
decimalLongitude	The geographic longitude (in decimal degrees, using the spatial reference system given in geodeticDatum) of the geographic centre of a Location. Positive values are east of the Greenwich Meridian, negative values are west of it. Legal values lie between -180 and 180, inclusive.
geodeticDatum	The ellipsoid, geodetic datum or spatial reference system (SRS) upon which the geographic coordinates given in decimalLatitude and decimalLongitude are based. Recommended best practice is use the EPSG code as a controlled vocabulary to provide an SRS, if known. Otherwise use a controlled vocabulary for the name or code of the geodetic datum, if known. Otherwise use a controlled vocabulary for the name or code of the ellipsoid, if known. If none of these is known, use the value "unknown".
georeferenceSources	A list (concatenated and separated) of maps, gazetteers or other resources used to georeference the Location, described specifically enough to allow anyone in the future to use the same resources.
georeferenceRemarks	Notes or comments about the spatial description determination, explaining assumptions made in addition or opposition to those formalised in the method referred to in georeferenceProtocol.
identifiedBy	A list (concatenated and separated) of names of people, groups or organisations who assigned the Taxon to the subject. Recommended best practice is to separate the values in a list with space vertical bar space (|) .
dateIdentified	The date on which the subject was determined as representing the Taxon. Recommended best practice is to use a date that conforms to ISO 8601-1:2019.
taxonID	An identifier for the set of taxon information (data associated with the Taxon class). May be a global unique identifier or an identifier specific to the dataset.
scientificNameID	An identifier for the nomenclatural (not taxonomic) details of a scientific name.
scientificName	The full scientific name, with authorship and date information, if known. When forming part of an Identification, this should be the name in the lowest level taxonomic rank that can be determined. This term should not contain identification qualifications, which should instead be supplied in the identificationQualifier term.
nameAccordingTo	The reference to the source in which the specific taxon concept circumscription is defined or implied - traditionally signified by the Latin "sensu" or "sec." (from secundum, meaning "according to"). For taxa that result from identifications, a reference to the keys, monographs, experts and other sources should be given.
kingdom	The full scientific name of the kingdom in which the taxon is classified.
phylum	The full scientific name of the phylum or division in which the taxon is classified.
class	The full scientific name of the class in which the taxon is classified.
order	The full scientific name of the order in which the taxon is classified.
family	The full scientific name of the family in which the taxon is classified.
genus	The full scientific name of the genus in which the taxon is classified.
taxonRank	The taxonomic rank of the most specific name in the scientificName.
vernacularName	A common or vernacular name.
taxonRemarks	Comments or notes about the taxon or name.

### Data set 2.

#### Data set name

Darwin Core Archive Geohistorical plants occurrences database - Identification History Supplementary file (identification.txt)

#### Data format

Darwin Core Archive format.

#### Number of columns

7

#### Character set

UTF-8

#### Download URL

http://ipt.gbif.fr/archive.do?r=ei2p_exotic_plants_geohistorical_occurrences_database&v=1.4

#### Data format version

1.4

#### Description

The Darwin Core Identification History is an extension allowing multiple identification/determinations of species occurrences, particularly name spellings found in each original text. All identifications including the current one are listed, while the current should also be repeated in the occurrence core for simple access (Source: https://tools.gbif.org/dwca-validator/extension.do?id=dwc:Identification). All column labels and descriptions are from https://dwc.tdwg.org/terms/.

**Data set 2.** 

Column label	Column description
id	An identifier for the Occurrence linked to the occurrence.txt file (same as occurrenceID). It can be repeated as a foreign key here.
identificationID	A unique identifier corresponding to the name spelling reported as found in the original text. This is the primary key of this table.
dateIdentified	The date on which the subject was determined as representing the Taxon. Recommended best practice is to use a date that conforms to ISO 8601-1:2019. The date format here is YYYY (e.g. 1694).
scientificName	The full scientific name, with authorship and date information, if known. When forming part of an Identification, this should be the name in the lowest level taxonomic rank that can be determined. This term should not contain identification qualifications, which should instead be supplied in the identificationQualifier term.
nameAccordingTo	The reference to the source in which the specific taxon concept circumscription is defined or implied - traditionally signified by the Latin "sensu" or "sec." (from secundum, meaning "according to"). For taxa that result from identifications, a reference to the keys, monographs, experts and other sources should be given.
vernacularName	A common or vernacular name.
taxonRemarks	Comments or notes about the taxon or name.

## Additional information

**Maintenance and future work**

All data will be maintained by their creators. They will be progressively enriched by new data during the current EI2P-VALEEBEE research project and also during the projects that the team will continue to conduct on invasive plant species thereafter.

The dataset is already archived and published through GBIF:

https://www.gbif.org/dataset/345820cc-a0a8-4d76-b7eb-fba85b21ad08

It will be regularly updated and versioned through GBIF.

## Supplementary Material

0D6863E9-2F42-5B8C-8A15-23D7503C3CEC 10.3897/BDJ.10.e76283.suppl1 Supplementary material 1 Glossary of acronyms Data type Glossary. File: oo_561277.txt https://binary.pensoft.net/file/561277 Morgane Claudel, Emilie Lerigoleur, Cécile Brun, Sylvie Guillerme

D4081542-86F7-5A9F-96FE-F0D60F525922 10.3897/BDJ.10.e76283.suppl2 Supplementary material 2 The synonymy of the ten species (TAXREF v13 and historical sources) Data type Vernacular name identified in historical literature. File: oo_561276.txt https://binary.pensoft.net/file/561276 Morgane Claudel, Emilie Lerigoleur, Cécile Brun, Sylvie Guillerme

6EC1B170-62DA-5520-A6FE-8ED99A6FF074 10.3897/BDJ.10.e76283.suppl3 Supplementary material 3 List of historical sources consulted from which the dataset was produced Data type Historical sources consulted. File: oo_561272.txt https://binary.pensoft.net/file/561272 Morgane Claudel

## Figures and Tables

**Table 1.** FAIRness assessment criteria used for this dataset.

**FAIR principles**	**FAIRness assessment criteria used**
FINDABLE	Manfrini Use a DOI for the dataset attributed by GBIF. Use unique identifiers (UUID) for each observation occurrence. Make persistent metadata and datasets thanks to the deposit on the GBIF platform. Use the Ecological Metadata Language (EML) internationally recognised standard to describe the database metadata and its associated projects, including standardised search keywords. Use a versioning system to allow future updates. Manfrini
ACCESSIBLE	Manfrini Data storage in GBIF in accordance with the guidelines for quality standards (e.g. use of EML). The GBIF repository provides efficient, rich services for various uses and users. Manfrini
INTEROPERABLE	Manfrini Standard vocabularies used as far as possible for some fields. Thesaurus used to search keywords from international thesauruses, such as GEMET including INSPIRE themes. Exclusive use of Darwin Core terms. A Darwin Core Archive offers a stable, straightforward and flexible framework for compiling biodiversity data from varied and variable sources (source: https://www.gbif.org/en/darwin-core). Manfrini
REUSABLE	Manfrini The Darwin Core Archive facilitates the reusability of the dataset because it enables publication in the GBIF. This compact package (a ZIP file) contains interconnected text files and enables users to share their data using a common terminology (source: https://www.gbif.org/en/darwin-core). Use an open format for the dataset (OpenDocument.ods) and open source software to reuse it. EML metadata includes provenance for raw and derived data. This data paper explains the data processing steps, curation protocol, quality assurance processes, methods and tools that permit long-term integrity and understandability of data. The spatial/temporal/taxonomic coverage is clearly mentioned in the EML metadata and in this data paper, as well as the CC-BY licence and rules for large reuse. Manfrini

**Table 2.** Phylogenetic classification of the studied taxa ordered by order, family, genus and species, with their number of occurrences and their hyperlink to the subsample by species on gbif.org.

**Order**	**Family**	**Genus**	**Species**	**Common name**	**Number of occurrences (%)**
Asterales	Asteraceae	*Helianthus*	*Helianthus tuberosus*	Jerusalem artichoke	**17 (8.5)**
Caryophyllales	Polygonaceae	*Reynoutria*	*Reynoutria japonica*	Japanese knotweed	**21 (10.6)**
Ericales	Balsaminaceae	*Impatiens*	*Impatiens glandulifera*	Indian balsam	**53 (26.6)**
Fabales	Fabaceae	*Robinia*	*Robinia pseudoacacia*	False-acacia	**26 (13.1)**
Fagales	Betulaceae	*Alnus*	*Alnus incana*	Grey alder	**7 (3.5)**
	Fagaceae	*Castanea*	*Castanea sativa*	Sweet chestnut	**7 (3.5)**
Lamiales	Scrophulariaceae	*Buddleja*	*Buddleja davidii*	Butterfly-bush	**17 (8.5)**
Rosales	Rosaceae	*Prunus*	*Prunus cerasifera*	Cherry plum	**20 (10.1)**
			*Prunus laurocerasus*	Cherry laurel	**20 (10.1)**
		*Spiraea*	*Spiraea japonica*	Japanese spiraea	**11 (5.5)**
